# Assessment of radiographic parameters in diagnosing discoid lateral meniscus: A retrospective MRI‐based study

**DOI:** 10.1002/jeo2.70564

**Published:** 2025-11-14

**Authors:** Jiawen Fong, Khai Cheong Wong, Merrill Lee, Xunqi Cheow, Joyce Suang Bee Koh, Tet Sen Howe, Meng Ai Png

**Affiliations:** ^1^ Department of Orthopaedic Surgery Singapore General Hospital Singapore Singapore

**Keywords:** discoid lateral meniscus, lateral joint space, magnetic resonance imaging, radiograph, parameters

## Abstract

**Purpose:**

It is difficult to diagnose discoid lateral meniscus (DLM) clinically, and various imaging modalities are employed to aid diagnosis. However, the use of radiographic parameters as a diagnostic tool remains unvalidated. Hence, this study aims to determine the validity of radiographic parameters and their role in diagnosing DLM.

**Methods:**

This is a retrospective study of magnetic resonance imaging (MRI)‐diagnosed DLM. MRI films were reviewed, and the presence of DLM was identified (Group A). A control group of age‐and gender‐matched patients with no reported abnormalities in their knees on MRI was identified (Group B). X‐ray parameters for all patients were measured, including squaring of the lateral femoral condyle (LFC), lateral joint space (LJS), cupping of lateral tibial plateau (CLTP), lateral tibial obliquity (LTO), fibula head height (FHH), height of the lateral intercondylar spine (LIS) and LFC notching, with normalised ratio (NR) values obtained for LJS and FHH by dividing their respective values against the interepicondylar distance (IED). Data analysis was performed to compare differences between both groups.

**Results:**

A total of 72 patients (36 in each group) were included. Our study showed that Group A had significantly smaller squaring of LFC (*p* = 0.012), wider LJS (*p* < 0.01), less CLTP (*p* < 0.01) and reduced LTO (*p* < 0.01). However, these same parameters were not significant in historical studies. There were no significant differences in the remaining parameters between Groups A and B. In addition, as compared to historical studies that show significant differences in LJS and FHH when comparing normal menisci versus DLM, our present study did not show a significant difference in the latter parameter.

**Conclusion:**

Widened LJS is a consistent X‐ray parameter seen in DLM. Inconsistencies in the significance of other radiographic parameters should drive caution when using them for diagnosis, and other imaging modalities should be utilised to confirm the presence of DLM.

**Level of Evidence:**

Level III.

**Abbreviations**
CLTP: depth of cupping of lateral tibial plateau
DLM: discoid lateral meniscus
FHH: fibula head height
IED: notching and interepicondylar distance
KS: kurtosis and skew
LFC: lateral femoral condyle
LIS: height of lateral intercondylar spine
LJS: lateral joint space
LTO: lateral tibial obliquity
MRI: magnetic resonance imaging
NR: normalised ratio
XR: X‐rays

## INTRODUCTION

Discoid lateral meniscus (DLM), an anatomical anomaly of the lateral knee meniscus, was first described by Young in 1889 [6]. The incidence of DLM is estimated to be 1%–15% of the population, and it is more commonly observed in Asian populations [2, 13]. Individuals with DLM may be asymptomatic but can also present with various symptoms such as pain, effusion, giving way, limitations in range of motion, atraumatic popping or snapping and clicking or locking [15, 17]. Depending on the posterior attachment and the degree of tibial plateau coverage, DLM can be divided into complete, incomplete and Wrisberg types [12].

DLM is diagnosed using magnetic resonance imaging (MRI), X‐rays (XR) of the affected knee joint and can also be confirmed arthroscopically. MRI is the imaging gold standard. However, traditionally, the use of radiographs is still relevant as an initial screening tool during initial presentation to our orthopaedic specialist clinics, as they are lower in cost and accessible even in the lower‐resource care settings. Kim et al. [14] evaluated parameters on plain radiographs of knees and found that historical XR features such as fibula head height (FHH) and lateral joint space (LJS) positively correlated with arthroscopically proven DLM. Other historical radiographic features that poorly correlated with DLM included: hypoplasia of the lateral femoral condyle (LFC), effusion, early osteoarthritis, cupping of the lateral aspect of the tibial plateau (CTP) and obliquity of the lateral tibial plateau (LTO) [8, 9]. However, current literature shows a lack thereof explanations or hypotheses for the correlation of certain XR parameters and DLM.

A review of the current landscape describes DLM diagnosed through imaging and/or arthroscopy that have undergone intervention [3, 9, 12, 23] but does not include asymptomatic DLM patients, potentially leading to selection bias in previous studies. Our study, however, utilises MRI‐diagnosed asymptomatic DLMs or the inclusion of normal knees without other pathologies to reduce selection bias. Hence, this study aims to evaluate the validity of these radiographic parameters in both symptomatic and asymptomatic patients. We hypothesise that XR features of DLM are historical and are not accurate in diagnosing the presence or absence of DLM.

## MATERIALS AND METHODS

A retrospective review of MRI scans performed in our institution was conducted. MRI protocol included sagittal thin‐section proton density, sagittal T2, coronal T1, coronal fat‐saturated T2 and axial T2‐weighted sequences in MRI 1.5 and 3.0 Tesla field strength machines. DLM was diagnosed based on coronal images with a minimum width at the meniscal body measuring 15 mm or more. We searched through our database of knee MRI scan reports for the keywords ‘discoid lateral meniscus’ between 2014 and 2017 to ensure uniform imaging protocols and radiology reporting standards within the institution during that timeframe. All MRI scans were reported by fellowship‐trained radiologists. We included both complete and incomplete DLM (Group A) and excluded skeletally immature patients (less than 16 years of age) and patients without available plain radiographs of the ipsilateral knee. To ensure minimal XR changes of the knee when the MRI was performed, a cut‐off of 6 months before or after the MRI was an acceptable range for the knee XR to be performed. This is to maximise the sample size in a retrospective study, as MRI waiting times in our institution may take up to 6 months to be scheduled, while acknowledging that major radiographic features of DLM are unlikely to change significantly over this duration in skeletally mature patients. Anterior–posterior and lateral views of the XRs were utilised, performed in the erect position with standard film‐focal distance of 1.15 m or 115 cm. We also included a control group (Group B) matched for age and gender, which consisted of randomly selected patients with normal MRI scans obtained from retrospective MRI knee studies with radiological report indicators labelled as ‘Normal’, that were reported by trained radiologists. Exclusion criteria for the DLM group included XR findings of severe osteoarthritis, previous knee surgery or other concomitant injuries.

**Figure 1:**
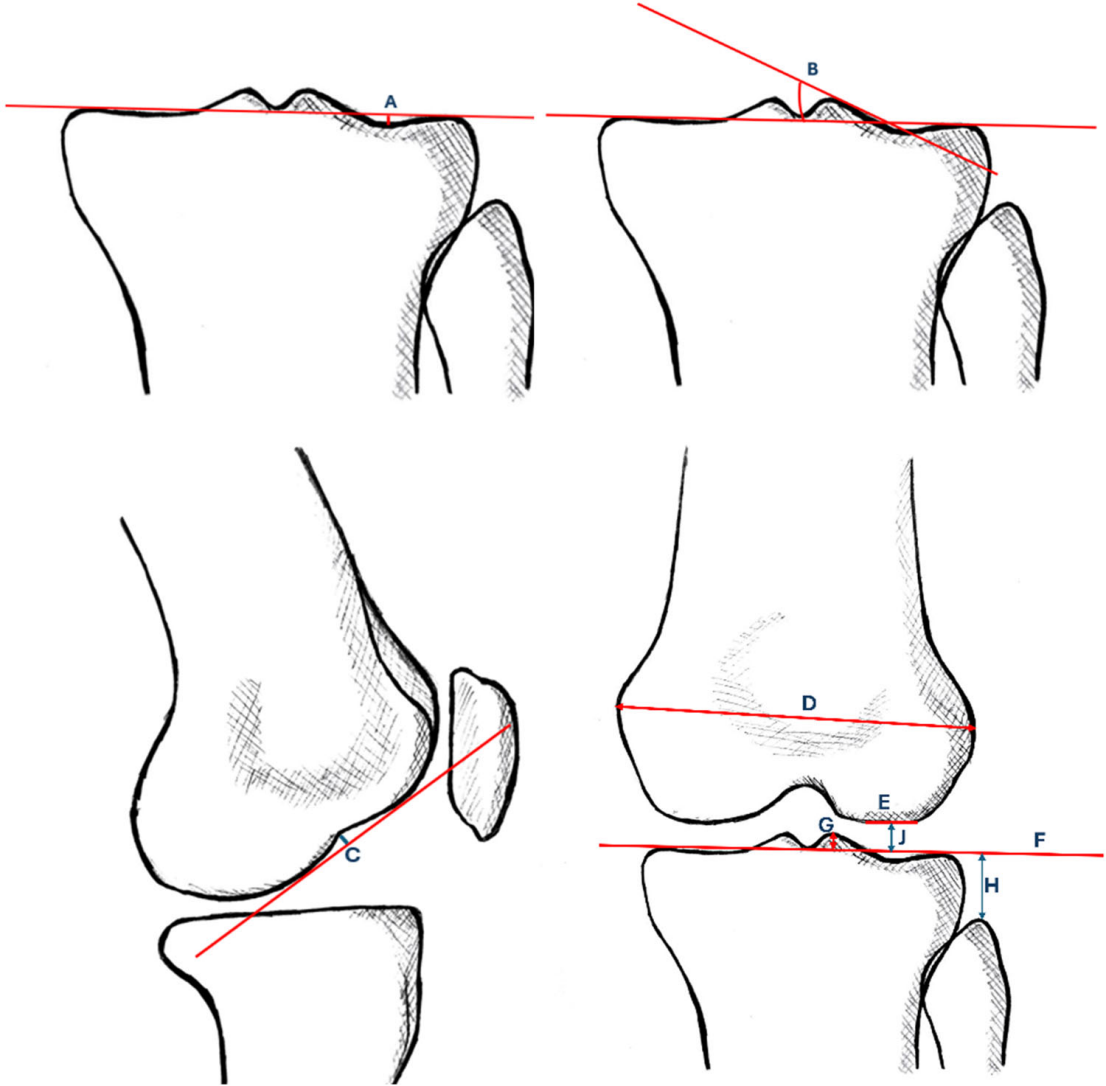
(Top left) Diagram showing distance A, measuring the degree of cupping of the lateral tibial condyle by using an imaginary line drawn at the level of the tibial joint line; (top right) diagram showing B, measuring the lateral tibial obliquity angle, measuring by taking the angle between the imaginary line drawn across the tibial joint line and the best‐fit line along the lateral tibial slope; (bottom left) diagram showing C, measuring the notching of the lateral femoral condyle (LFC) by measuring the distance from the inner‐most notch of the LFC to the tangential line to the LFC; (bottom right) diagram showing D, measuring the interepicondylar distance of the femur; E, measuring the extent of squaring of the LFC which is taken as the flat portion of the articular surface of the LFC; F, which is the imaginary line drawn at the level of the tibial joint line; G, measuring the height of the lateral intercondylar spine by taking the distance between F and the highest‐most point of the lateral tibial spine; H, measuring the height of the fibula head by taking the distance between F and the top‐most point of the fibula head; and J, measuring the lateral joint space, taken by measuring the distance between F and the surface of the LFC.

**Figure 2:**
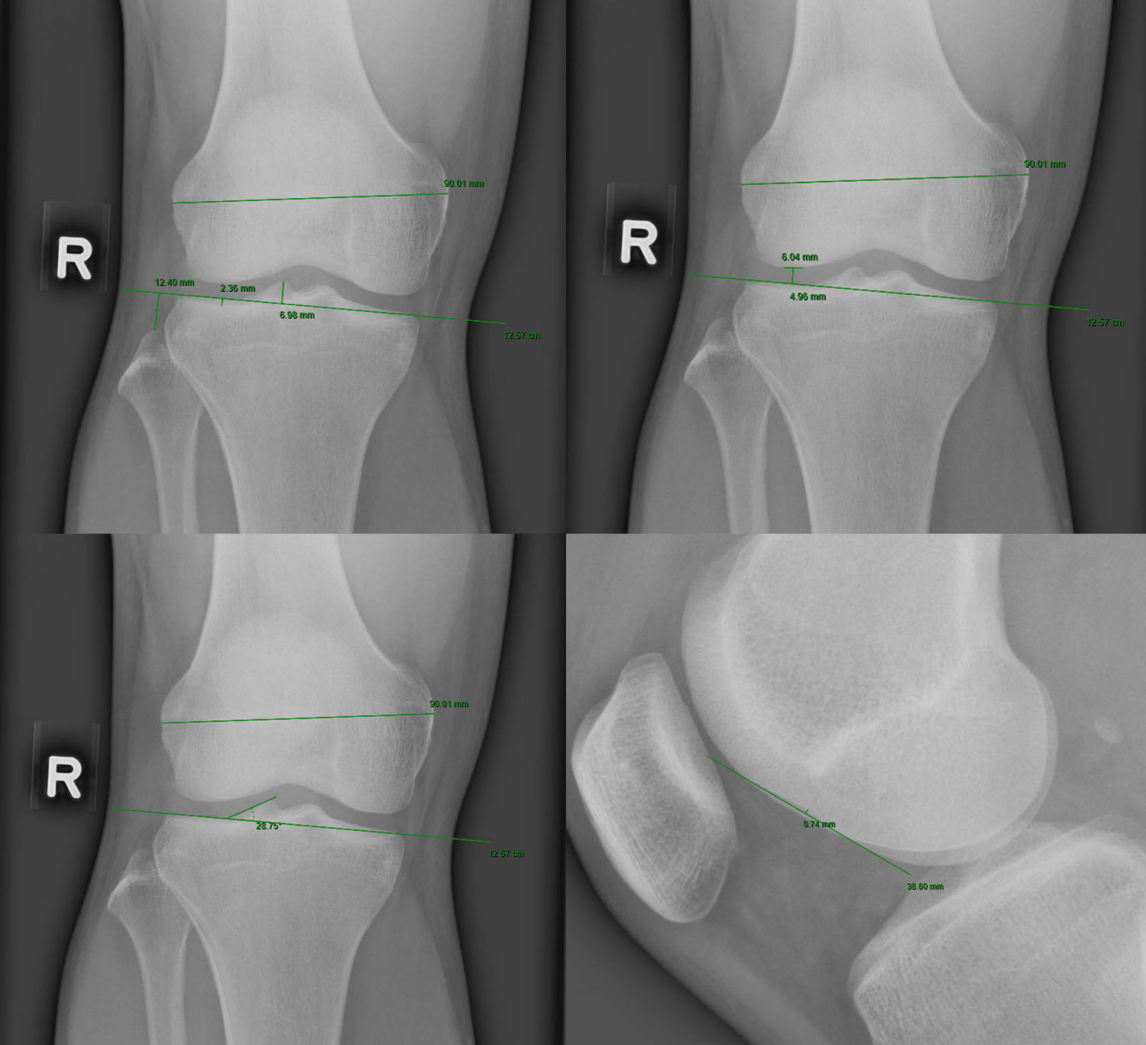
(Top left) Measurements of IED, imaginary tibial joint line, LIS, FHH and CLTP; (top right) measurements of squaring of LFC and LJS; (bottom left) showing how the LTO is measured; (bottom right) LFC notching. CLTP, depth of cupping of lateral tibial plateau; FHH, fibula head height; IED, interepicondylar distance; LFC, lateral femoral condyle; LIS, height of lateral intercondylar spine; LJS, lateral joint space; LTO, lateral tibial obliquity.

All measurements were performed on the PACS clinical viewer (VueMotion) using in‐built measurement tools as opposed to a physical transparent ruler with 1/10 mm precision used by Kim et al. [14]. Figure 1 illustrates how the measurements were made on the radiographs to determine the following parameters: extent of squaring of the length of the straight lateral femoral articular surface, LJS, depth of cupping of lateral tibial plateau (CLTP), lateral tibial obliquity (LTO), FHH, height of lateral intercondylar spine (LIS), LFC notching and interepicondylar distance (IED). Normalised ratio (NR) values were obtained for LJS and FHH by dividing the respective value against each patient's IED to reduce discrepancy in knee dimensions as per Kim et al. [14]. Cut‐off values used, as described in historical literature [14], included: more than 10 mm squaring of the LFC, lateral femorotibial joint space of more than 5 mm, more than 1 mm cupping of the LTP, height of fibular head of less than 13 mm from the tibial joint line, as well as LIS of less than 6 mm. Intraobserver reliability was checked by repeating the measurements for all 72 radiographs at a 1‐week interval, and was accepted if the value lies within a range of 0.5 mm. Interobserver reliability was assured by repeating the measurements of all 72 radiographs by three independent observers and ensuring the values measured were of an acceptable range (within 0.5 mm). The average of all the values taken at a 1‐week interval was then averaged. Figure 2 shows the measurements done using the radiographs.

The data were analysed using SPSS software. For raw values, Shapiro–Wilk test with *p* value < 0.05 to reject the null hypothesis of normal distribution was first used for normality assessment of the data (Table 1). Raw data that did not lie in normal distribution were then subjected to a Mann–Whitney *U* test corrected for Bonferroni to determine its significance with a *p* value of 0.05. The remainder of the raw data that fell within normal distribution were subjected to a two‐tailed *t*‐test corrected for Bonferroni to determine their significance, with a *p* value of 0.05. Categorical data based on the criteria in the previous paragraph were subjected to Pearson's *χ* ^2^ test corrected for Bonferroni with an *α* value of 0.00556 (nine variables involved). Effect sizes for all data were obtained using Cohen's *d* and Cramer's *V* for continuous and categorical data, respectively. Confidence intervals (CIs) at 95% were obtained for the data. Receiver operating characteristic (ROC) curves are also obtained for the individual variables with specificity and sensitivity values.

**Table 1:** Shapiro–Wilk test.

Variables	*p* value	
	Discoid group	Normal group
Extent of squaring of LFC	0.010	0.037
LJS	0.149	0.631
LJS (NR)	0.104	0.046
CLTP	0.004	0.008
LTO	0.387	0.257
FHH	0.247	0.624
FHH (NR)	0.532	0.091
Height of LIS	0.785	0.587
LFC notching	0.002	0.000

Abbreviations: CLTP, depth of cupping of lateral tibial plateau; FHH, fibula head height; LFC, lateral femoral articular surface; LFC, lateral femoral condyle; LIS, height of lateral intercondylar spine; LJS, lateral joint space; LTO, lateral tibial obliquity; NR, normalised ratio.

## RESULTS

We analysed 36 patients in Group A and 36 age‐sex‐matched patients in Group B. Table 2 shows the measurements of the various radiographic features in our study cohort. Table 3 shows the CIs for the respective continuous parameters.

**Table 2:** Measurements of the various radiographic features in the study cohort.

	**Group A**	**Group B**	**Effect sizes**	***p* value corrected for Bonferroni** ^a^
Mean age	46.4 ± 15.2	46.31 ± 15.18	—	NA
Males	22 (61.1%)	22 (61.1%)	—	
Females	14 (38.9%)	14 (38.9%)	—	
Complete discoid	29 (80.6%)	—	—	
Incomplete discoid	7 (19.4%)	—	—	
Extent of squaring of LFC (mm)	6.29 ± 3.34	8.47 ± 3.82	−0.425	0.0107
Squaring of LFC > 10 mm (*N*)	5 (13.9%)	13 (36.1%)	0.257	0.029
LJS (mm)	6.39 ± 1.21	4.76 ± 1.29	1.31	<0.01
LJS > 5 mm (*N*)	32 (88.9%)	15 (41.7%)	0.496	<0.001
LJS (NR)	0.074 ± 0.013	0.0539 ± 0.015	−0.830	<0.01
LJS (NR) > 0.06 (*N*)	29 (80.6%)	15 (41.7%)	0.399	<0.001
CLTP (mm)	0.85 ± 0.80	1.66 ± 1.09	−0.539	0.001
CLTP > 1 mm (*N*)	13 (36.1%)	25 (69.4%)	0.334	0.005
LTO (°)	30.5 ± 4.93	35.93 ± 5.86	0.997	<0.001
LTO > 17.6° (*N*)	36 (100%)	36 (100%)	—	NA
FHH (mm)	12.4 ± 3.55	13.94 ± 4.15	0.391	0.102
FHH < 13 mm (*N*)	22 (61.1%)	18 (50.0%)	0.112	0.343
FHH (NR)	0.142 ± 0.037	0.160 ± 0.0471	0.006	0.081
FHH NR < 0.153 (*N*)	23 (63.9%)	16 (44.4%)	0.195	0.098
LIS (mm)	7.92 ± 1.42	7.83 ± 1.62	0.064	0.786
LIS < 6 mm (*N*)	2 (5.6%)	4 (11.1%)	0.101	0.394
LFC notch height from lateral view (mm)	0.32 ± 0.31	0.378 ± 0.338	−0.112	0.502321
LFC notch height from lateral view > 1 mm (*N*)	1 (2.8%)	1 (2.8%)	0.000	1
Total number	36 (100%)	36 (100%)		

*Note*: Cohen's *d* utilised for continuous variables, and Cramer's *V* utilised for categorical variables.

Abbreviations: CLTP, depth of cupping of lateral tibial plateau; FHH, fibula head height; LFC, lateral femoral articular surface; LFC, lateral femoral condyle; LIS, height of lateral intercondylar spine; LJS, lateral joint space; LTO, lateral tibial obliquity; NA, not applicable; NR, normalised ratio.

^a^ Bonferroni correction for continuous variables was performed using SPSS software; for categorical data, *p* value is determined by calculating the adjusted *p* value (of 0.05) divided by the total number of variables [9].

**Table 3:** Confidence intervals of the respective parameters.

	Confidence interval
Extent of squaring of LFC (mm)	0.489 to 3.86
LJS (mm)	−2.22 to −1.05
LJS (NR)	−0.025 to −0.012
Cupping of LTP (mm)	0.358 to 1.26
LTO (°)	2.85 to 7.94
FHH (mm)	−0.307 to 3.32
FHH (NR)	−0.003 to 0.038
LIS (mm)	−0.813 to 0.617
LFC notch height from lateral view (mm)	−0.0939 to 0.211

Abbreviations: CLTP, depth of cupping of lateral tibial plateau; FHH, fibula head height; LFC, lateral femoral condyle; LIS, height of lateral intercondylar spine; LJS, lateral joint space; LTO, lateral tibial obliquity.

Group B has a significantly larger LFC (8.47 ± 3.82 vs. 6.29 ± 3.34 mm, CI: 0.489–3.86, *p* value = 0.012), with a larger proportion of patients meeting the cut‐off of >10 mm, which was not significant (13.9% vs. 26.1%, *p* value = 0.029 > 0.00556).

Group A has a significantly larger LJS (6.39 ± 1.21 vs. 4.76 ± 1.29, CI: −2.22 to −1.05, *p* value ≤ 0.01) with a significantly larger number of patients meeting the cut‐off of >5 mm (88.9% vs. 41.7%, *p* value < 0.01). Group A also has a significantly larger LJS NR (0.074 ± 0.013 vs. 0.0546 ± 0.0145, CI: −0.255 to −0.123, *p* value < 0.01) with a larger proportion of patients meeting the cut‐off of >0.06 (80.6% vs. 41.7%, *p* value < 0.01).

Group A has a significantly smaller CLTP (0.85 ± 0.80 vs. 1.66 ± 1.09, CI: 0.358–1.26, *p* value ≤ 0.01) with a significantly smaller number of patients meeting the cut‐off of >1 mm (36.1% vs. 69.4%, *p* value < 0.01).

Group A has a significantly smaller LTO (30.5 ± 4.93 vs. 35.93 ± 5.86, CI: 2.85–7.94, *p* value < 0.01). However, all patients of both groups met the cut‐off of >17.6° (100% vs. 100%, *p* value = not applicable).

Table 4 shows the ROC, area under the curve (AUC), specificity and sensitivity values. Only LJS (AUC = 0.830), LJS (NR) (AUC = 0.835) and LIS (AUC = 0.518) have an AUC of >0.5, but with a sensitivity of 0.917, 0.722 and 0.694, respectively. However, only LJS showed a Youden Index close to 1, at 0.473, with a suggested cut‐off for LJS at 4.75 mm instead of 5 mm.

**Table 4:** ROC values with specificity and sensitivity.

Variables	Area under the curve	Cut‐off value	1‐ Specificity	Sensitivity	Youden Index (YI)	ROC curves
Extent of LFC squaring >10 mm	0.325	14.1	0.111	0.056	−0.833	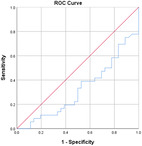
LJS > 5 mm	0.830	4.76	0.556	0.917	0.473	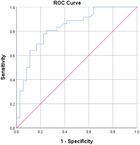
LJS NR > 0.06	0.835	0.0650	0.167	0.722	−0.111	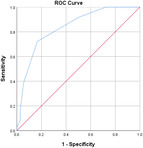
CLTP > 1 mm	0.279	1.24	0.500	0.306	−0.194	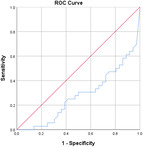
LTO > 17.6 deg	0.233	44.3	0.056	0.0208	−0.932	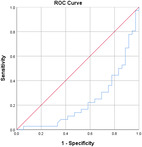
FHH < 13 mm	0.385	17.5	0.167	0.111	−0.722	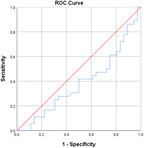
FHH NR < 0.153	0.392	0.185	0.167	0.167	−0.666	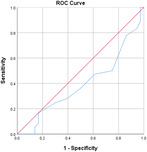
LIS < 6 mm	0.518	7.10	0.667	0.694	0.361	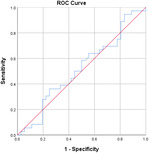
LFC notch >1 mm	0.454	0.400	0.333	0.417	−0.250	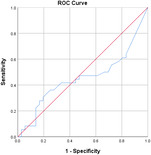

Abbreviations: CLTP, depth of cupping of lateral tibial plateau; FHH, fibula head height; LFC, lateral femoral condyle; LIS, height of lateral intercondylar spine; LJS, lateral joint space; LTO, lateral tibial obliquity; NR, normalised ratio; ROC, receiver operating characteristic.

## DISCUSSION

The most significant findings of this present study that we found in DLM patients include XR features of smaller LFC, widened LJS and its NR values, decreased CLTP and a smaller LTO. We compared this to significant historical XR parameters based on Kim et al. [14], which showed a widened LJS and a higher FHH. This differs from our study, which shows significant findings in the former but not the latter features.

LJS appears to be consistently widened across various studies in the presence of DLM [10, 14, 16]. The meniscus covers completely or almost completely over the lateral tibial plateau [7] in DLM, and this is associated with increased thickness in the lateral knee joint [11]. The knee is hence more likely to go into a varus than a valgus inclination [19]. In the normal knee, 40% of the weight is transmitted via the lateral compartment [22], and we postulate that this may be less in DLM in view of its thickness and size with a varus tilt, thus resulting in a widened LJS. In our study, using the values obtained from calculating AUC, sensitivity and specificity, LJS thus appears to be reliable as one of the parameters that can be used on XR for the diagnosis of DLM. However, the suggested cut‐off point for LJS was 4.75 mm instead of the 5 mm suggested in historical literature. It is important to note that statistical significance may not translate well to clinical significance, as a difference of 1–2 mm on XRs may be easily overlooked. This can result in an over‐ or underestimate of patients with DLM and caution should still be applied when using the LJS as a parameter.

There are several other parameters that we found to be significant in our study but not in historical parameters [12], such as smaller LFC, decreased CLTP and a smaller LTO on XR films. LTO was included as a measurement in our study as it was traditionally found to be one of the factors that were seen on radiographs in DLM [14]. A literature review of the current landscape reveals no known cause or potential reasons for the aforementioned three factors seen on XRs in DLM. These parameters appear to be inconsistent across various studies [10, 12], and caution should be taken when using these parameters as a means for diagnosing DLM on XR. In addition, a possible reason for the difference in results in the LTO in our present study as compared to that in Kim et al. [14] could potentially be attributed to the lack of a proper definition of how it should be measured. Kim et al used the definition of the angle formed by the imaginary joint line and articular line of the lateral tibial plateau without fully defining the start and end points of the latter. In contrast, our study uses the angle between the best‐fit tangential line along the lateral tibial slope and the imaginary joint line. This best‐fit line involves marking the start and end points of the tibial slope, then further marking eight points along the surface equidistant apart, followed by drawing a best‐fit line through the points. By using this method of measuring the LTO, we aim to reduce the inconsistency by using fixed landmarks to make it more reproducible. However, despite the use of such a method, our results showed insignificance in the LTO values, and thus, invalidating its use despite a more standardised measurement.

A parameter that is significant in other studies [10, 12, 16] but not in this present study includes a high FHH. We postulate that the discrepancies from previous studies could be a result of differences in, but not limited to, measurement techniques or the software used, patient selection criteria or inclusion of asymptomatic cases. However, its significance is not diagnostic of DLM [12]. A study by Song et al. [21] describes XR features of complete DLM, which has a lower FHH and larger LJS as does in incomplete DLM. It is thus difficult to differentiate between complete and incomplete DLM, and normal knees on XRs, as both complete and incomplete DLM can potentially appear to have non‐significant XR parameters and go undiagnosed. The finding by Song et al also contradicts the findings in the aforementioned other studies of a high fibula head that is usually present in DLM. The inconsistent findings of high FHH, thus, may not be adequate as an XR parameter to prove the presence of DLM and should be used with caution.

Historic parameters that are not significant both in other studies [10, 12, 16] and the present study include the degree of notching of the LFC and LIS. The aforementioned factors are not well‐studied in current literature with little known about its correlation to DLM. Thus, we propose that they should not be used as a diagnostic tool for DLM on XRs.

While some studies have shown that XR parameters may prove useful as a screening tool [20], several other studies have determined that supposed XR features had little value in the detection of DLM [1, 5], as in the case of meniscal injuries. DLM is associated with an increased incidence of meniscal tears due to its differences in anatomical morphology [13]. The presence of meniscal tears may affect XR features and also obscure the identification of DLM on MRI [18], making MRI less sensitive. A study by Asik et al. [4] has also shown the incongruence in clinical and radiological findings with arthroscopic results, and there is higher value of arthroscopic evaluation for DLM. Therefore, adjuncts such as arthroscopy, which allows for direct visualisation of the meniscus, or alternative methods of MRI or three‐dimensional reconstruction can be used to delineate the meniscus better to mitigate the insufficiency of XRs in diagnosing DLM.

The usage of MRI in our study to select patients allows for a wider selection pool to include both asymptomatic and symptomatic DLM, as well as normal menisci to reduce the selection bias of patients included. To the best of our knowledge, our present study is the first to analyse nonarthroscopic, incidental MRI‐proven DLM patients and compare it to MRI‐proven normal knees [20, 21]. This is as opposed to a recent study by Jiang et al. [10], who compared 60 patients each with MRI‐proven normal meniscus but with other concomitant pathologies, with MRI‐proven DLM. He found significant differences in a higher FHH, widened LJS, decreased LTO and increased CLTP. He also included another proposed XR parameter measuring the distance between the lateral tibial intercondylar spine and lateral femoral condyle (DLC), which proved significant. While significant XR parameters analysed by Jiang et al. were almost similar to those in our study, the results may have been skewed by the state of the knees included in his study, as normal menisci with other knee pathologies were not excluded in his control group. The discordance in the finding of high FHH also remains unanswered.

Limitations of our study include the small study cohort, but this is limited by the low incidence of DLM. This is a retrospective study and inherently subject to biases typical of observational studies such as selection bias. In addition, a separate study involving the measurements by involving the other authors or other surgeons not involved in the present study may help provide additional validation to inter‐rater variabilities. Another limitation of our study is the small number of incomplete DLM patients, and hence meaningful statistical subgroup analysis is not feasible.

## CONCLUSION

Radiographs have limited and inconsistent diagnostic value, and thus larger prospective studies are required to determine their significance in the diagnosis of DLM. While widened LJS appears to be a sensitive parameter for DLM, the presence of this XR finding cannot exclude other abnormalities in the knee, and other imaging adjuncts should still be utilised for a complete diagnosis. In addition, XR parameters may also vary in different study populations and hence further prospective studies should be performed to determine appropriate cut‐off values that may be useful as diagnostic criteria for DLM.

## AUTHOR CONTRIBUTIONS

All authors contributed to the study conception and design. Material preparation, data collection and analysis were performed by all authors. The first draft of the manuscript was written by Jiawen Fong, and all authors commented on previous versions of the manuscript. All authors read and approved the final manuscript.

## CONFLICT OF INTEREST STATEMENT

The authors declare no conflicts of interest.

## ETHICS STATEMENT

The authors have nothing to report.
